# Micro actions in colorectal cancer screening participation: a population-based survey study

**DOI:** 10.1186/s12885-015-1465-9

**Published:** 2015-05-29

**Authors:** Siu Hing Lo, Jo Waller, Charlotte Vrinten, Christian von Wagner

**Affiliations:** Cancer Research UK Health Behaviour Research Centre, Department of Epidemiology and Public Health, University College London, Gower Street, London, WC1E 6BT UK

**Keywords:** Colorectal cancer screening, Behaviour, Procrastination, Planning, Self-regulation

## Abstract

**Background:**

Low uptake of colorectal cancer (CRC) screening is a cause for concern. This study explored people’s anticipated response to receiving the test kit to shed light on past screening uptake and help inform future interventions to increase participation.

**Methods:**

Face-to-face interviews were conducted with respondents living in England who were eligible for CRC screening as part of a population-based ‘omnibus’ survey. Respondents were asked what they would do (‘micro actions’) if they received a CRC screening test kit through the mail (apart from completing it or not), and their unprompted responses were coded (multiple codes allowed). Past ‘ever’ uptake and screening intention were also recorded. The final analysis included 1237 respondents aged 60–70.

**Results:**

Respondents who said that they would decide after some thought’ (p < .001), ‘put [it] aside to deal with later’ (p < .001), ‘put it on the “to do list/ pile”’ (p < .05) or ‘discuss it with a health care professional’ (p < .01) had decreased odds of having participated. Those who said they would ‘read the instruction leaflet’ (p < .001), ‘put the kit near the toilet’ (p < .001) or ‘decide when to do the test’ (p < .05) were more likely to have taken part in CRC screening. With the exception of ‘decide when to do the test’ and ‘discuss it with a health care professional’, all associations with past uptake remained significant after adjusting for other micro actions and screening intention. ‘Make a note somewhere (to remind myself)’ was mentioned by less than 1 % of respondents.

**Conclusions:**

Delay-causing and preparatory micro actions were associated with past CRC screening uptake. Self-regulatory micro actions (e.g. making a note to remind oneself) were rarely mentioned as responses to receiving a screening invitation. Interventions aimed at reducing delay and facilitating preparatory and self-regulatory behaviours might help increase uptake. The behaviour-focused survey method is a promising avenue for future health behaviour research.

## Background

Randomised controlled trials have demonstrated that regular, repeated screening using the Faecal Occult Blood test (FOBt) reduces colorectal cancer (CRC) mortality by up to 25 % [1, 2]. In England, the National Health Service (NHS) Bowel Cancer Screening Programme (BCSP) sends all age-eligible (60–74 year-old) men and women a free FOBt kit on a biennial basis. However, uptake is only around 54 % in any one screening invitation round [3], although some non-responders (~13 %) participate when re-invited two or four years later [4].

Previous research on CRC screening uptake has examined the role of social cognitive factors derived from psychological models (e.g. Health Belief Model, Theory of Planned Behaviour) [5]. CRC screening is perceived to be a ‘good idea’ by a large majority [6], and many indicate they intend to participate [7]. However, social cognitive factors are more strongly associated with intention than with actual behaviour [8, 9]. The behavioural sequence leading up to cancer screening participation is a less well-explored terrain. The present study therefore aimed to explore the association between ‘micro actions’, behaviours preceding the behaviour of interest, to explain cancer screening participation. Another aim was to examine whether any associations between micro actions and cancer screening behaviour were independent of screening intention. If so, this would suggest that micro actions can explain behaviour above and beyond known social cognitive factors.

There are two main categories of micro action that may be important in understanding screening behaviour. Firstly, there are delay-causing behaviours, such as procrastination, which would be expected to inhibit test completion. In a recent survey, around 16 % of non-responders gave procrastination as the main reason for non-participation in CRC screening [10]. Another study of NHS screening records also showed that late response to one or more previous screening invitations was a risk factor for non-response to subsequent invitations [11].

The second category can be conceptualised as behaviours involved in the planning of or preparation for test completion, and would be expected to be positively associated with participation, by helping people translate a positive intention into actual behaviour. ‘Implementation intentions’ are an example of a planning intervention method aimed at helping people to do this [12]. The aim is to help people plan and thus overcome any self-regulatory problems such as procrastination [13]. The method has been successfully applied to a wide range of health behaviours [14]. Nevertheless, there is substantial heterogeneity in study findings, which has been largely attributed to the level of guidance offered to participants [15]. Previous interventions using pre-formulated implementation intentions to promote CRC screening using FOBt have had no overall impact on screening uptake in a large, population-based sample [16], and only a modest impact in a sample of previous responders [17]. A better understanding of relevant micro actions could therefore be used to improve the guidance offered through planning or other behaviour change interventions to promote CRC screening uptake.

The present study examined micro actions in the context of CRC screening in England. The first aim of the study was to obtain a picture of the behaviours undertaken between the moment an individual receives a CRC screening test kit and (non-) completion of the test. An open-ended question format was used to allow hitherto unknown micro actions to be identified and to avoid response bias. Following this, we examined which micro actions were associated with previous participation in CRC screening. It was hypothesised that micro actions suggestive of procrastination would be associated with lower past uptake, while planning-related and other micro actions contributing to test completion would be associated with a higher rate of past uptake.

## Methods

Data were collected as part of a TNS Research International population-based omnibus survey conducted in Great Britain between January and March 2014. Up to 4000 adults per week are interviewed in Great Britain for the omnibus survey. The TNS omnibus survey defines sample points using 2001 Census small-area statistics and the Postcode Address File (stratified by social grade and Government Office Region) which are used for random location sampling selection. Response rates are not recorded. However, at each location, quotas are set for age, gender, children in the home and working status to ensure a balanced sample. Survey respondents are verbally requested to volunteer their participation in face-to-face interviews using computer-assisted personal interviewing (CAPI). Three doors are left between each successful interview. NHS ethics approval was obtained for this study (13/NW/0707).

### Participants

Only respondents living in England who were aged 58–70 years and had no history of CRC were included in the cancer screening section of the omnibus survey (n = 1568). Respondents aged 58–59 (n = 187) were excluded from the present analysis because at the time of the interview they were not yet eligible for CRC screening through the English NHS BCSP. Therefore, all included respondents should all have been invited for CRC screening through the organised national programme, irrespective of whether they believed they had been invited or not. Responses to related questions were compared, and cases were excluded if responses were logically inconsistent with each other (n = 78; e.g. responding ‘immediately decide not to do the test’ if receiving a test kit AND ‘will do the test’ when sent one). Respondents who had missing values (i.e. ‘refused’ or ‘don’t know’) for screening uptake (n = 66) were also excluded. The exclusions resulted in a final sample of 1237 respondents.

### Measures

The term ‘bowel cancer’ was used throughout the survey, as it is commonly used to refer to colorectal cancer in the United Kingdom.

### Micro actions

Respondents were informed at the beginning of the survey that the subsequent part would consist of questions about bowel cancer screening and the home-based stool test, which is offered through the NHS BCSP. They were shown images of the invitation letter, the information booklet, the instruction leaflet and the screening test kit to ensure they recalled the test. They were then asked the open-ended question:

‘We are interested in what people do, or would do, when they are sent the screening test kit. Imagine you are at home and you have just opened an envelope in which you find a test kit and a leaflet with instructions of how to complete the test. Of course, you would usually need to wait until you have a bowel motion before you can complete the kit. By bowel motion we mean “to go for a poo”. What would you do after you receive the test kit through the post?’

Pilot interviews (n = 8) had been conducted earlier to develop predefined answer categories. These categories were then tested in a pilot online survey (n = 427), in which respondents were shown all the answer categories as well as having the option to give a verbatim response.

In contrast to the pilot survey, respondents in the present study gave open replies to the interviewers. Responses were coded by the interviewers using answer categories that were not shown to the respondents. Verbatim responses were recorded if interviewers were unsure or if the answer did not fit into any of the codes. Interviewers were instructed to collect as much information as possible and use multiple codes as appropriate:

- Immediately decide not to do the test/ throw the kit away [excluded from analysis]
- Immediately decide to do the test/ do the test [excluded from analysis]
- Decide (whether or not to do) the test after some thought
- Put (the kit) aside to deal with later
- Put on my’things to do list/ pile’
- Verify source of information
- Read the instruction leaflet
- Discuss it with a health care professional
- Discuss it with my partner/family/friend
- Decide when to do the test
- Make a note somewhere (e.g. diary, post-it, calendar to remind myself)
- Put the kit near the toilet (so that I have it ready when I have a bowel motion)
- Other, namely… [verbatim response]

Since the research aim was to examine micro actions that are potentially undertaken between receiving the test kit and (non-) completion of the test kit, responses referring to screening intention and the actual behaviour were excluded. As a result, the response categories ‘immediately (decide) to do the test’ and ‘immediately decide not to do the test’ were not further analysed. Intention formation (e.g. ‘decide whether or not to do the test after some thought’), however, was considered a micro action. Verbatim responses (n = 48) were coded by two independent coders (SHL and CV), with 82 % agreement and a kappa inter-rater agreement of 0.78, suggesting there was substantial inter-rater agreement [18, 19]. Coding disagreements were resolved through discussion. Most ‘other’ responses were recoded under the existing answer categories. Two new categories were created following the coding process: ‘unclear’ (n = 11) and ‘other micro action’ (n = 6). Due to the small number of cases, these newly created categories were not included in the analysis.

For each micro action, a dichotomous variable was created to indicate either that a respondent had mentioned the micro action (1) or not (0). Respondents who had not mentioned any micro actions had a score of (0) for all micro actions but were retained in the analysis.

### Past screening uptake

Respondents were then asked if they had ever been invited to do a stool test for the National Health Service (NHS) Bowel Cancer Screening Programme (BCSP). If their answer was affirmative, they were asked further questions to determine the number of times they had been invited and the number of times they had participated in the screening programme. A dichotomous variable for past ever screening uptake was created with the categories non-responders (never invited or never completed) and responders (≥1 test kits completed).

### Screening intention

Respondents were asked ‘Do you think you will do the stool test when you are (next) sent one?’ on a five-point scale (no, definitely not [1]/no, probably not [2]/not sure [3]/yes, probably [4]/yes, definitely [5]/not applicable [missing]). Screening intention was included as a control variable in a multivariable regression analysis.

### Sociodemographics

Sex, age, marital status (married/ widowed, divorced or separated/ single), ethnicity (white/ non-white) and social grade were recorded to describe the sample. The National Readership Survey social grade classification system based on occupation (or previous occupation if retired) was used as a measure of social grade: AB (managerial/ professional); C1 (supervisory); C2 (skilled manual), DE (semi-skilled/ unskilled manual, state pensioners, casual/ lowest grade workers or unemployed). For people who were not working, the chief wage earner in the household’s occupational status was used.

### Data analysis

Descriptive statistics were first given for each micro action and each micro action by past screening uptake. Correlations between micro actions were also examined. Simple logistic regression analysis was then used to examine bivariate associations between past uptake and endorsement of each micro action. Micro actions that showed a statistically significant association at the .05 level and screening intention were included in multivariable logistic regression analyses. All analyses used pairwise deletion and were conducted with Stata Version 13SE [20].

## Results

### Sample characteristics

Of the total included sample (n = 1237), 50.4 % were men. The age range of the sample was 60–70 with a mean age of 65.1 (SD = 3.2). Most respondents (65.2 %) were married, 26.4 % were widowed, divorced or separated, and 8.5 % were single. All social grades were represented in the sample, with 26.0 % in grades AB, 21.8 % in C1, 18.4 % in C2 and 33.7 % in DE. In line with the low prevalence of ethnic minorities among older age groups in the national population of England [21], only 3.9 % (n = 48) of respondents were non-white.

### Micro actions

Overall, roughly half (49.7 %, n = 615) of the respondents mentioned at least one relevant micro action. Nearly half (48.5 %, n = 600) responded that they would ‘immediately (decide to) do the test’. A small minority (9.0 %, n = 111) would ‘immediately decide not to do the test’. These responses were not further analysed because they did not fit our definition of a micro action. Only one-fifth (21 %, n = 149) of the respondents who had answered that they would either immediately do or not do the test also mentioned a relevant micro action.

The number and proportion of respondents mentioning each micro action are presented in Table 1. Over a quarter (27.2 %) of respondents would ‘read the instruction leaflet’, 10.8 % would ‘put the kit near the toilet’, 9.9 % would ‘put [it] aside to deal with later’, 8.1 % would ‘decide when to do the test’ and 5.4 % would ‘decide after some thought’. Other micro actions were mentioned by fewer respondents: 3.2 % would ‘discuss it with their partner/ family/ friend’, 2.4 % would ‘put [it] on [their] things to do list/ pile’, 1.1 % would ‘discuss it with a health care professional’, 0.7 % would ‘verify the source of information’ and 0.7 % would ‘make a note somewhere (to remind themselves)’.

**Table 1:** Number of people mentioning each micro action (n = 1237)

What would you do after you receive the test kit?	Micro action % (*n*)
Read the instruction leaflet	27.2 % (337)
Put the kit near the toilet	10.8 % (134)
Put aside to deal with later	9.9 % (122)
Decide when to do the test	8.1 % (100)
Decide after some thought	5.4 % (67)
Discuss it with partner/family/friend	3.2 % (40)
Put on ‘things to do list/pile’	2.4 % (30)
Discuss it with health care professional	1.1 % (13)
Verify source of information	0.7 % (9)
Make a note somewhere	0.7 % (9)

Table 2 shows the correlations between micro actions, which were generally weak or non-significant. The highest correlation was between ‘decide when to do the test’ and ‘read the instruction leaflet’ (r = 0.26, p < .001).

**Table 2:** Correlation matrix of micro actions

	Read the instruction leaflet	Put the kit near the toilet	Put aside to deal with later	Decide when to do the test	Decide after some thought	Discuss it with partner/family/friend	Put on ‘things to do list/ pile’	Discuss it with health care professional	Verify source of information	Make a note somewhere
Read the instruction leaflet	-									
Put the kit near the toilet	0.15	-								
Put aside to deal with later	−0.07**	−0.05	-							
Decide when to do the test	0.26***	0.04	−0.06*	-						
Decide after some thought	0.01	−0.05	−0.02	−0.01	-					
Discuss it with partner/family/friend	0.16***	0.10***	0.05	0.08**	0.02	-				
Put on ‘things to do list/ pile’	−0.01	−0.02	0.04	0.07*	−0.01	0.03	-			
Discuss it with health care professional	0.00	−0.01	−0.01	−0.00	−0.02	0.12***	−0.02	-		
Verify source of information	0.08**	0.00	−0.03	0.08**	0.02	0.04	−0.01	0.08	-	
Verify source of information	0.08**	0.12***	0.00	0.15***	0.02	0.09	0.05	−0.01	−0.01	-

* *p* < .05;**p < .01; ***p < .001.

### Micro actions and past uptake

Of the total included sample, 70.1 % indicated they had responded to at least one screening invitation. Of the 29.9 % who had never screened, 51.9 % indicated they had never been invited. Bivariate analyses showed that the following micro actions were associated with decreased odds of past uptake: ‘put aside to deal with later’ (54.9 % uptake among those who mentioned it vs. 71.8 % for those who did not, p < .001), ‘decide after some thought’ (49.3 % vs. 71.3 %, p < .001), ‘put on my things to do list’ (46.7 % vs. 70.7 %, p < .01) and ‘discuss it with a health care professional’ (38.5 % vs. 70.4 %, p < .05). Micro actions associated with increased odds of past ever screening uptake were: ‘read the instruction leaflet’ (82.2 % vs. 65.6 %, p < .001), ‘put the kit near the toilet’ (94.0 % vs. 67.2 %, p < .001) and ‘decide when to do the test’ (83.0 % vs. 69.0 %, p < .01; Tables 1 and 3).

**Table 3:** Micro actions by past uptake and logistic regression results of micro actions as explanatory variables of past screening uptake

	Past uptake	Bivariate results		Multivariable results, Adjusted for micro actions		Multivariable results, Adjusted for micro actions and screening intention	
	Row % (n)	Unadjusted OR	95 % CI	Adjusted OR	95 % CI	Adjusted OR	95 % CI
Total	70.1 % (1237)						
Read the instruction leaflet							
Did not mention	65.6 % (900)	1.00 (ref.)		1.00 (ref.)		1.00 (ref.)	
Mentioned	82.2 % (337)	2.43***	1.78-3.31	2.05***	1.47-2.86	1.70*	1.11-2.61
Put the kit near the toilet							
Did not mention	67.2 % (1103)	1.00 (ref.)		1.00 (ref.)		1.00 (ref.)	
Mentioned	94.0 % (134)	7.69***	3.72-15.90	6.38***	3.06-13.29	3.00**	1.35-6.69
Put aside to deal with later							
Did not mention	71.8 % (1115)	1.00 (ref.)		1.00 (ref.)		1.00 (ref.)	
Mentioned	54.9 % (122)	0.47***	0.33-0.70	0.53**	0.36-0.79	0.54*	0.33-0.88
Decide when to do the test							
Did not mention	69.0 % (1137)	1.00 (ref.)		1.00 (ref.)		1.00 (ref.)	
Mentioned	83.0 % (100)	2.20**	1.29-3.76	1.64	0.92-2.91	1.20	0.62-2.33
Decide after some thought							
Did not mention	71.3 % (1170)	1.00 (ref.)		1.00 (ref.)		1.00 (ref.)	
Mentioned	49.3 % (67)	0.39***	0.24-0.64	0.38***	0.22-0.63	0.45*	0.25-0.83
Discuss it with partner/family/friend							
Did not mention	70.3 % (1197)	1.00 (ref.)		-		-	
Mentioned	62.5 % (40)	0.70	0.37-1.35				
Put on ‘things to do list/pile’							
Did not mention	70.7 % (1207)	1.00 (ref.)		1.00 (ref.)		1.00 (ref.)	
Mentioned	46.7 % (30)	0.36**	0.18-0.75	0.35**	0.16-0.74	0.26**	0.11-0.63
Discuss it with health care professional							
Did not mention	70.4 % (1224)	1.00 (ref.)		1.00 (ref.)		1.00 (ref.)	
Mentioned	38.5 % (13)	0.26*	0.09-0.81	0.21*	0.07-0.69	0.28	0.06-1.27
Verify source of information							
Did not mention	70.2 % (1228)	1.00 (ref.)		-		-	
Mentioned	55.6 % (9)	0.53	0.14-1.99				
Make a note somewhere							
Did not mention	70.0 % (1228)	1.00 (ref.)		-		-	
Mentioned	77.8 % (9)	1.50	0.31-7.24				
Screening intention (1–5)				-		4.56***	3.74-5.57

* *p* < .05;**p < .01; ***p < .001.

In the multivariable models including all significant micro actions and/or screening intention, all but two micro actions remained independently associated with past ever screening uptake (Table 3). ‘Decide when to do the test’ was the only micro action that was no longer significantly associated with past uptake after adjusting for other micro actions (OR = 1.64, 95 % CI: 0.92-2.91, n.s.). This suggests it was a micro action performed in conjunction with other micro actions, in particular ‘read the instruction leaflet’ (r = 0.26, Table 2). ‘Discuss it with a health care professional’ was no longer significantly associated with past uptake after adjusting for screening intention (OR = 0.28, 95 % CI: 0.06-1.27, n.s.), suggesting its association with uptake is not independent of intention. In other words, intending to speak to a health care professional might reflect uncertainty about whether to do the test or not, although the results should be interpreted with caution due to the low numbers who endorsed this micro action. ‘Decide after some thought’ (OR = 0.45, 95 % CI: 0.25-0.83, p < .05), ‘put aside to deal with later’ (OR = 0.54, 95 % CI: 0.33-0.88, p < .05) and ‘put on my things to do list/ pile’ (OR = 0.26, 95 % CI: 0.11-0.63, p < .01) remained negatively associated with past uptake after adjusting for other micro actions and intention. ‘Read the instruction leaflet’ (OR = 1.70, 95 % CI: 1.11-2.61, p < .05) and ‘put the kit near the toilet’ (OR = 3.00, 95 % CI: 1.35-6.69, p < .01) also remained associated with higher past uptake.

## Discussion

This study is the first to examine micro actions related to completing a CRC screening stool test kit, and their association with past screening uptake. As expected, micro actions causing delay in test completion (e.g. ‘put aside to deal with later’, ‘put on “things to do list/pile”’, ‘decide after some thought’) were negatively associated with past uptake. Micro actions related to preparation for and planning of test completion (e.g. ‘read the instruction leaflet’, ‘put the kit near the toilet’) were positively associated with past uptake. These associations were independent of other micro actions and screening intention, suggesting they could at least partially explain why levels of intention to take part in CRC screening are often higher than actual participation. The finding that roughly half of age-eligible non-responders indicated they had not been invited, also suggests factors other than intention and social cognitive factors influence screening participation. In the context of the English national screening programme, it is unlikely that eligible non-responders had truly not been invited; it is more likely that they had failed to notice or forgotten the mailed invitation.

‘Decide when to do the test’ was associated with past uptake, but the association was not independent of other micro actions. This suggests it is a micro action which tends to be performed in conjunction with other micro actions. ‘Discuss with a health care professional’ was also negatively associated with past uptake. However, this association was not independent of screening intention, perhaps due to health conditions for which CRC screening is contraindicated or uncertainty about the benefits of screening in general. The default, home-based screening process in England does not involve direct contact with General Practitioners or other health professionals. Only patients with special concerns would be expected to discuss screening with a health professional.

This study adds to a growing body of evidence about the role of procrastination and other delay-causing behaviours in CRC screening. While previous research showed that delay predicts lower subsequent screening participation [11], the present study showed that delay-causing micro actions were associated with screening uptake independent of screening intention. Tackling causes of delay such as procrastination therefore seems an important goal for future interventions aimed at promoting uptake among positive intenders.

‘Put the kit near the toilet’ was associated with the highest level of screening uptake (94 %) compared with all other micro actions. Interestingly, however, only a small minority (11 %) actually mentioned doing this. Prompting people to put the kit near the toilet might therefore promote uptake among positive intenders who might not have done this of their own accord. Having said that, a previous pre-formulated implementation intentions intervention, which among other things suggested putting the kit near the toilet, had no overall effect on uptake [16]. However, the intervention delivery mode (a mailed leaflet) used in that study might have compromised the effect of the intervention. Future research should examine if different intervention delivery modes or tailoring can make more effective use of such prompts. A more recent study reported more encouraging results of a computer-tailored implementation intentions intervention aimed at increasing CRC screening in a US population using colonoscopy, sigmoidoscopy or FOBt [22].

Perhaps surprisingly, few of the micro actions endorsed in this study were behaviours that would be defined as self-regulatory behaviours, which are often viewed as the key mechanisms through which planning interventions help translate intentions into behaviour [13, 23]. In particular, micro actions related to goal monitoring and responding to discrepancies between intention and behaviour were almost never mentioned. For example, ‘make a note somewhere to remind myself’ was mentioned by less than 1 % of respondents. This suggests that there is potential for self-regulatory processes to reduce the so-called ‘intention-behaviour gap’ in the context of CRC screening. Alternatively, it might indicate that these would not resonate well with the public.

A strength of this study was the use of omnibus survey methodology, which reduces participation bias associated with the topic of the survey. In addition, we used an open-ended question to generate survey responses that could shed light on people’s behaviour as opposed to their beliefs. Open-ended questions are less prone to priming people with answers than closed-ended questions. However, the methodology likely contributed to the very small numbers of endorsements for some micro actions and might have led to the opposite problem, under-reporting of micro actions, particularly among those who said they would immediately decide to do the test and were possibly not probed further about their immediate behaviour. Future research may need to address this limitation by increasing the total sample size and comparing results from closed-ended and open-ended questions. Differences in endorsement rates between open-ended and close-ended questions could be used to examine under- or over-reporting of micro actions.

The present study also placed the open-ended question at the beginning of the survey to avoid attempts to match responses to self-reported past screening uptake. The study was nevertheless limited by its cross-sectional design. Screening uptake was measured retrospectively, while micro actions were framed in terms of the hypothetical present. Examining the predictive value of micro actions for cancer screening participation and other health behaviours in longitudinal studies would be a valuable pursuit for future research.

## Conclusions

Micro actions were associated with past uptake of CRC screening among a population-based sample in England. Interventions aimed at preventing delay and promoting preparatory and self-regulatory micro actions might improve uptake. Future research should test the merits of behaviour-focused survey measures in the context of other health behaviours.
